# Multiple Alternative Splicing and Differential Expression Pattern of the Glycogen Synthase Kinase-3β (*GSK3β*) Gene in Goat (*Capra hircus*)

**DOI:** 10.1371/journal.pone.0109555

**Published:** 2014-10-15

**Authors:** Yuguo Hou, Yilin Wang, Yan Wang, Tao Zhong, Li Li, Hongping Zhang, Linjie Wang

**Affiliations:** Institute of Animal Genetics and Breeding, College of Animal Science and Technology, Sichuan Agricultural University, Chengdu, Sichuan, P.R. China; University of Valencia, Spain

## Abstract

Glycogen synthase kinase-3β (GSK3β) has been identified as a key protein kinase involved in several signaling pathways, such as Wnt, IGF-Ι and Hedgehog. However, knowledge regarding GSK3β in the goat is limited. In this study, we cloned and characterized the goat *GSK3β* gene. Six novel *GSK3β* transcripts were identified in different tissues and designated as *GSK3β1, 2, 3, 4, 5* and *6*. RT-PCR was used to further determine whether the six *GSK3β* transcripts existed in different goat tissues. Bioinformatics analysis revealed that the catalytic domain (S_TKc domain) is missing from GSK3β2 and GSK3β4. GSK3β3 and GSK3β6 do not contain the negative regulatory sites that are controlled by p38 MAPK. Furthermore, qRT-PCR and western blot analysis revealed that all the *GSK3β* transcripts were expressed at the highest level in the heart, whereas their expression levels in the liver, spleen, kidney, brain, *longissimus dorsi* muscle and uterus were different. These studies provide useful information for further research on the functions of *GSK3β* isoforms.

## Introduction

Glycogen synthase kinase-3 (GSK3) is a serine/threonine kinase that is mainly regulated by phosphorylation of its target substrates or itself. Since its initial purification from rabbit skeletal muscle [1], GSK3 has been continuously identified in connection with multiple pathways and shown to be a key component in the regulation of over fifty diverse proteins [2], [3]. GSK3 plays a crucial role in the regulation of cell fate, regulating processes such as embryonic development, cell proliferation and apoptosis [4]–[6]. Furthermore, GSK3 has been linked to many human diseases such as cancer [7], Alzheimer's disease [8], [9] and type II diabetes [10].

In mammals, GSK3 is primarily generated from two known genes: *GSK3α* and *GSK3β*. Interestingly, *GSK3α* is not found in birds [11]. GSK3 contains a two-domain kinase fold consisting of a β-strand domain at the N-terminus and an α-helical C-terminal domain [12]. The two isoforms have 98% sequence identity in the catalytic domain. GSK3α is 5 kDa larger than GSK3β due to a glycine-rich amino-terminus [13]. Phosphorylation of Ser9 and Ser21 causes inactivation of GSK3β and GSK3α, respectively [14], [15], while activation of GSK3β and GSK3α is dependent on the phosphorylation of Tyr216 and Tyr279, respectively [16]. Although studies have indicated that the two GSK3 isoforms are functionally redundant [17], other studies have shown that they have different functions in the regulation of transcriptional activation [4]. In cardiac tissue, the two isoforms have different activities in response to pressure overload [18] and in mediating the differentiation of murine bone marrow-derived mesenchymal stem cells into cardiomyocytes [19].

Two alternative splice variants of *GSK3β,* named *GSK3β1* and *GSK3β2,* have been isolated from human and mouse tissues [20], [21]. Alternatively spliced mRNAs significantly contribute to protein diversity. It has been shown that mutations causing abnormal splicing are associated with disease [22]; for example, the mis-splicing of *GSK3β* resulted in the emergence of leukemia stem cells [23]. The isoforms of *GSK3β* have distinct substrate preferences [24] and phosphorylation activity on neural-associated proteins [25]. Thus, *GSK3β2* has been recognized as a neuron-specific isoform [26]. Our previous study identified five transcripts in pig tissues and showed that *GSK3β5* exhibits differential effects on glycogen synthesis in PK-15 cells [27].

Most studies on GSK3 have been carried out in humans and mice, but information on the goat *GSK3β* gene is still limited. Alternative splicing of *GSK3β* has been identified in many animal models but not in domestic animals. In this study, we cloned and characterized the goat *GSK3β* gene and identified six novel *GSK3β* splice variants in various goat tissues.

## Results

### Characterization of the goat GSK3β gene

The *GSK3β* cDNA sequence of sheep was compared with mouse, porcine and human sequences, and specific primer pairs were designed in the conserved regions to amplify fragments covering the entire putative coding sequence of the goat *GSK3β1* gene. The goat *GSK3β1* gene is 1334 bp in length (GenBank Acc. No.: KJ649149) and consists of a 1263 bp open reading frame that encodes a 420 amino acid protein with an expected molecular weight of 46.72 kDa and an isoelectric point (pI) of 8.68. The amino acid sequence encoded by *GSK3β1* shares 100%, 99%, 99% and 99% sequence identity with sheep (NP_001123212.1), mouse (NP_062801.1), porcine (AFN70426.1) and human *GSK3β1* (NP_001139628.1), respectively.

**Figure 1:**
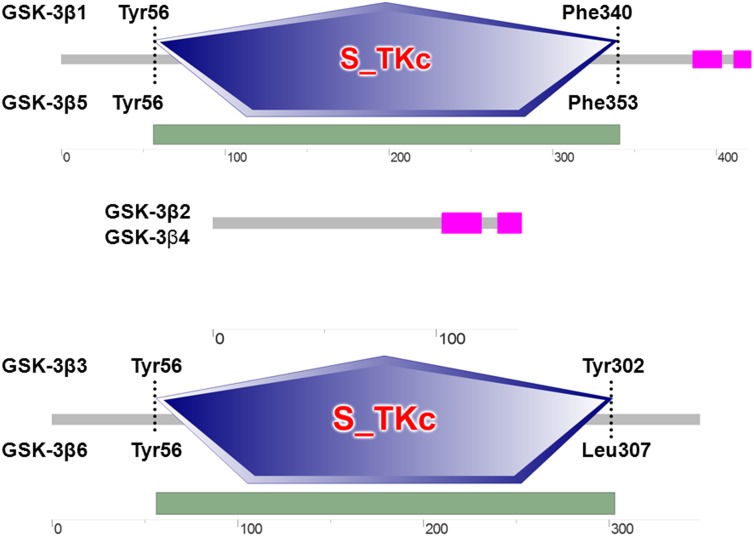
Schematic comparisonof goat GSK3β isoforms. The domain structure was analyzed by or using the online software SMART. GSK3β1, GSK3β3, GSK3β5 and GSK3β6 have the S_TKc domain structure that plays important roles in many cellular activities mediated by protein kinases and phosphoprotein phosphatases. The blast of GSK3β2 and GSK3β4 sequences result in no significant character to identify from established cutoffs.

Protein structure and function predictions indicate that goat GSK3β has a 285 aa S_TKc domain between Tyr^56^ and Phe^340^ (Fig. 1), which is recognized as a catalytic domain of the serine/threonine protein kinase family. At the C-terminal region of the predicted protein, two regions of low compositional complexity were predicted by the SEG program: one from Ala^386^ to Ala^402^ and another from Ala^411^ to Ser^420^.

### Identification of multiple alternative transcripts of goat GSK3β

**Figure 2:**
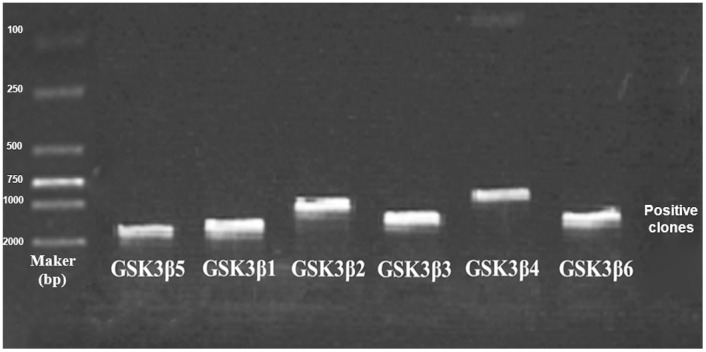
Identification and sequence of multiple alternative transcripts of goat GSK3β. We sequence more than 100 positive clones to identify the multiple *GSK3β* transcripts. Six variant transcripts of *GSK3β* have been shown in the picture.

**Figure 3:**
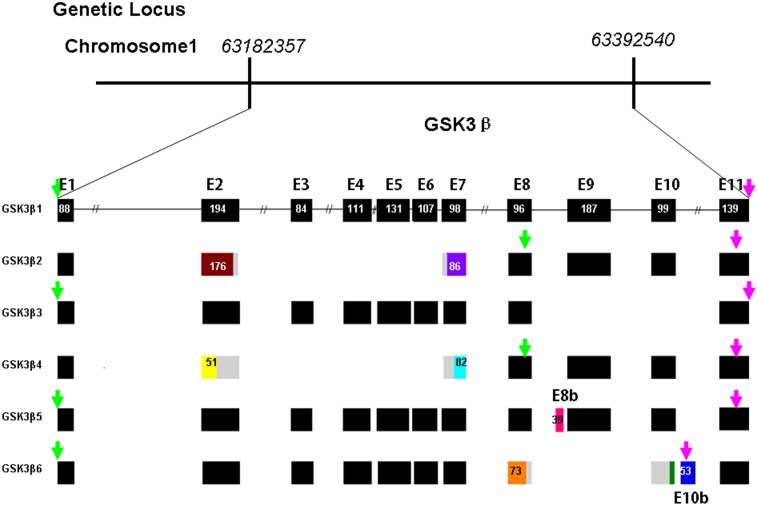
Schematic representation of the genomic and alternative splicing of goat *GSK3β*. BLAST searched the all goat nucleotide databases reveal that goat *GSK3β* gene was located on chromosome 1, the nucleotide sequence was corresponding to the genetic locus from 63182357 bp to 63392540 bp and consist of eleven exons. Green and pink arrows represent the beginning and ending site of open reading frame respectively. Various exons were distinguished by different color.

**Figure 4:**
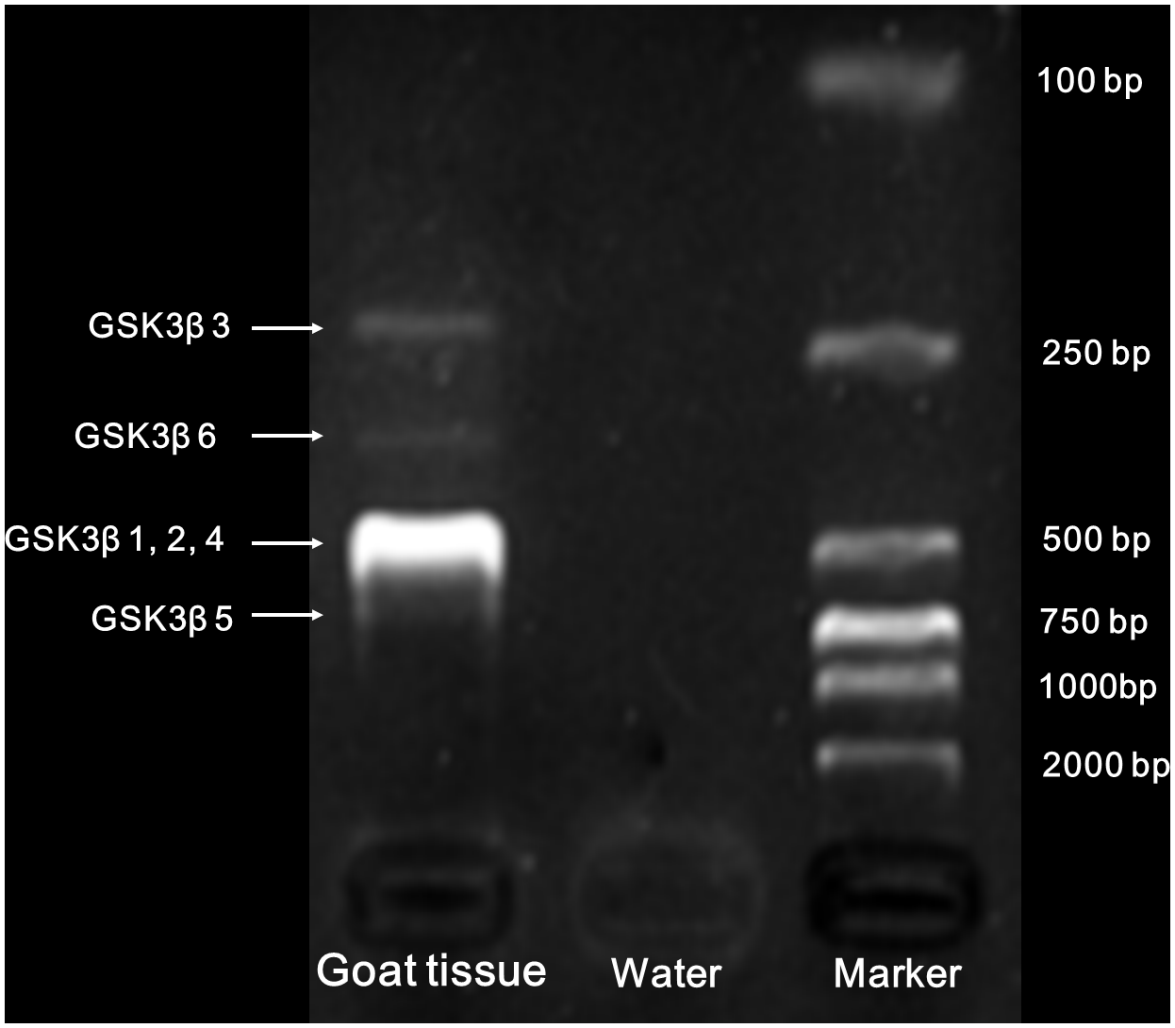
Detection of alternative transcripts of goat *GSK3β* by RT-PCR analysis. The difference in C-terminals changed the length of nucleotide sequences which were selected by primer pairs (Exon8-11-F and Exon8-11-R). The PCR products were separated by 2.5% agarose gel electrophoresis and transcripts of *GSK3β3*, *GSK3β5* and *GSK3β6* were visually present and sequencing identified.

In the process of cloning the goat *GSK3β* gene, we screened and sequenced more than 100 positive clones to identify the multiple *GSK3β* transcripts (Fig. 2). Six transcripts were observed and designated *GSK3β1*, *GSK3β2, GSK3β3, GSK3β4, GSK3β5* and *GSK3β6* (Fig. 3). The nucleotide sequences of each transcript are 1334, 871, 1048, 742, 1373 and 1095 bp in length, respectively. The cDNA sequences of the six goat *GSK3β* transcripts were deposited in GenBank as KJ649149–KJ649154. Based on the sequence alignment results, the main differences between the *GSK3β* splice variants were found between the seventh and eleventh exons. We amplified these variable regions using specific primers (Exon8-11-F and Exon8-11-R, Table 1), and the PCR products were separated by 2.5% agarose gel electrophoresis to visualize the different expression patterns. As shown in Fig. 4, purifying and sequencing the individual bands revealed that six transcripts exist in different goat tissues.

**Table 1:** Primer sequences used in this study.

Primer	Sequence(5′-3′)	Size	Tm(°C)	Location	Location
GSK3β-CDS	F:ATGTCAGGGCGGCCCAGAA	1360 bp	61.7	5′-UTR	cDNA Cloning
	R:GACCAGTGTTGCTGAGTGAC			3′-UTR	
GSK3β1	F:ATAGATGTATGGTCTGCAGGCTGTG	203 bp	62.0	Exon6-Exon7	qRT-RCR
	R:AAGACCTTAGTCCAAGGATGTGCCT			Exon8-Exon9	
GSK3β2	F:AAGATGGCAGCAAGGTAA	184 bp	58.0	Exon2*	qRT-RCR
	R:CAACACACAGCCTGCAATAC			Exon2*/Exon7*	
GSK3β3	F:TGGACTAAGATGCTAATGCTG	149 bp	60.5	Exon8/Exon11	qRT-RCR
	R:GACCAGTGTTGCTGAGTGAC			3′-UTR	
GSK3β4	F:ATGTCAGGGCGGCCCAGAA	230 bp	56.5	5′-UTR	qRT-RCR
	R:AACACACAGCCTGCCCAGGA			Exon2*/Exon7*	
GSK3β5	F:CACCAACAAGGGAGCAAAT	124 bp	58.5	Exon8	qRT-RCR
	R:CGCACTCCTGAGGTGAAAT			Exon8b	
GSK3β6	F:TGTTGGCTGAGCTGTTGCT	192 bp	55.7	Exon7	qRT-RCR
	R:ATGTCGGCAGGCATTCACT			Exon10b	
EXON8-11	F:CACCAACAAGGGAGCAAAT		61.2	Exon 8	RT-PCR
	R:GACCAGTGTTGCTGAGTGAC			3′-UTR	ASdetection
β-actin	F:CCTGCGGCATTCACGAAACTAC	87 bp	59.5		
	R:ACAGCACCGTGTTGGCGTAGAG				

*Incomplete exon region.

**Figure 5:**
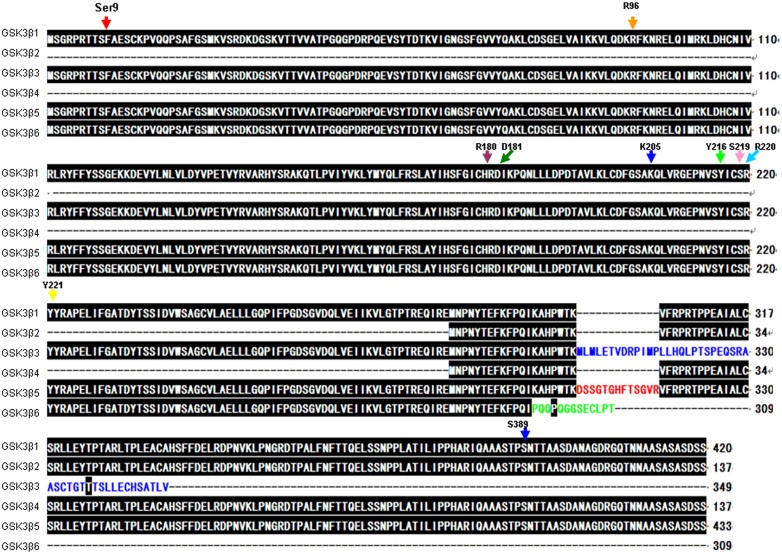
Amino acids sequences alignment of *GSK3β* isoforms in goat. Open reading frames of six transcripts were performed using NCBI ORF Finder. Important phosphorylation site of GSK3β have been marked by colorful arrows. The alternative splicing of GSK3β were named: GSK3β1, GSK3β2, GSK3β3, GSK3β4, GSK3β5 and GSK3β6, which encoded six proteins with 420, 137, 349, 137, 433 and 309 amino acids respectively. 13 amino acids were inserted into the GSK3β4 kinase domain. GSK3β3 and GSK3β6 contain two different C-terminuses.

The six goat *GSK3β* transcripts encode 420, 137, 349, 137, 433 and 309 amino acid proteins (Fig. 5). For consistency, all of the amino acid sequences were compared with GSK3β1. Because only a partial nucleotide sequence was extracted for *GSK3β2*, the amino acid sequence of GSK3β2 only contained the eighth, ninth, tenth and an incomplete eleventh exon (nucleotides 679 to 870 of the eleventh exon). Coincidentally, the same amino acid sequence was identified for GSK3β4. Both GSK3β2 and GSK3β4 have an expected molecular weight of 14.81 kDa and an isoelectric point of 7.28. GSK3β3 is 71 aa shorter than GSK3β2, with a molecular weight of 39.35 kDa and a theoretical isoelectric point of 8.47. Interestingly, *GSK3β5* contains 39 additional nucleotides inserted between exons 8 and 9, resulting in a 13 amino acid insertion in the kinase domain. GSK3β6 has a molecular weight of 34.86 kDa and a theoretical isoelectric point of 8.66. Remarkably, the additional exon (10b) in *GSK3β6* did not form a longer amino acid sequence but rather inhibited its transcription (Table 2).

**Table 2:** Sequence characterization of different goat GSK3β alternative transcripts.

Gene names	nt	Size protein (aa)	pI	Mw(kDa)	Accession numbers
GSK3β1	1334	420	8.68	46.72	KJ649149
GSK3β2	871	137	7.28	14.81	KJ649150
GSK3β3	1048	349	8.47	39.35	KJ649151
GSK3β4	742	137	7.28	14.81	KJ649152
GSK3β5	1373	433	8.69	47.99	KJ649153
GSK3β6	1095	309	8.66	34.86	KJ649154

Structures of the functional domains of multiple predicted GSK3β protein isoforms indicate that GSK3β1 has a 247 aa S_TKc domain between Tyr^56^ and Tyr^302^. Both GSK3β5 and GSK3β6 have the same domain between Tyr^56^ and Phe^353^ (298 aa) and between Tyr^56^ and Leu^307^ (252 aa). However, two low complexity regions were not found in GSK3β3 and GSK3β6. The GSK3β2 and GSK3β4 sequences were atypical. Because no domains were identified when they were subjected to a BLAST search of established functional domain structures (Fig. 1).

### Genomic structure of the goat GSK3β gene

To obtain more information about the genomic structure of the goat *GSK3β* gene, we searched the goat nucleotide database [28] by BLASTN and found a contig encoding the *GSK3β* cDNAs. The full-length coding sequence of the goat *GSK3β* gene is formed from eleven major exons and two minor exons, which are alternatively spliced to generate multiple *GSK3β* isoforms. The contig is located on chromosome 1, and the nucleotide sequence corresponds to the genetic locus from 63,182,357 bp to 63,392,540 bp. Compared to the transcript of *GSK3β1*, *GSK3β2* only contains the first, eighth, ninth, tenth, and eleventh exons, 176 nucleotides in the second exon and 86 nucleotides in the seventh exon. The altered transcript encodes a different open reading frame. The ninth and tenth exons have been deleted in *GSK3β3. GSK3β4* was formed without the third, fourth, fifth and sixth exons, 143 nucleotides in the second exon and 17 nucleotides in the seventh exon. *GSK3β5* contains a special 39-nucleotide insert called exon 8b that was originally situated in the intron between the eighth and ninth exons. *GSK3β6* lost the ninth exon, 23 nucleotides from the eighth exon and 82 nucleotides from the tenth exon; however, it contained an additional 53-nucleotide insert, which was named exon 10b (Fig. 3).

### Tissue expression patterns of GSK3β isoforms in the goat

qPCR was used to further assess the mRNA expression patterns of the goat *GSK3β* transcripts in different tissues. The isoform-specific primer pairs were designed as Fig. S1. The PCR fragments were purified and sequenced to confirm the correct amplification of the individual transcripts.

**Figure 6:**
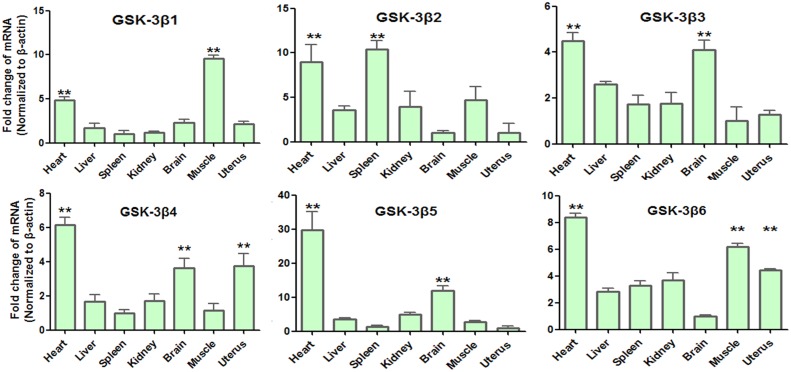
mRNA expression pattern in different tissues was performed by qRT-PCR. The samples represent goat heart, liver, spleen, kidney, brain, *longissimus dorsi* muscle, uterus respectively. The bars represent the mean ± SEM. (n = 3), and beta-actin was used as a control. Significant levels were analyzed by t-test. **P<0.01.

All transcripts were found to be predominantly expressed in heart (*P*<0.01), whereas expression in the liver and kidney was relatively weak. The *GSK3β1* gene was expressed at significantly higher levels in heart and *longissimus dorsi* muscle (*P*<0.01) than that in the other tissues examined. *GSK3β2* mRNA was found to be predominantly expressed in heart and spleen (*P*<0.01) with lower levels found in *longissimus dorsi* muscle. *GSK3β3* mRNA was found to be predominantly expressed in heart and brain (*P*<0.01) with lower levels found in the liver and little *GSK3β3* mRNA found in spleen and kidney. The *GSK3β4* and *GSK3β5* genes were expressed at the highest levels in heart and brain (*P*<0.01), whereas expression in the liver, spleen, kidney and *longissimus dorsi* muscle was relatively weak. *GSK3β6* mRNA was predominantly observed in heart, *longissimusdorsi* muscle and uterus (*P*<0.01) with lowest levels found in brain. Moreover, the mRNA expression levels in the uterus, *longissimusdorsi* muscle, spleen and brain varied, as *GSK3β4* and *GSK3β6* mRNA was abundant (*P*<0.01) in the uterus but *GSK3β2* and *GSK3β5* were barely expressed. *GSK3β1* and *GSK3β6* mRNA was abundant (*P*<0.01) in *longissimus dorsi* muscle but *GSK3β3*, *GSK3β4* and *GSK3β5*. The others were barely detected in spleen but *GSK3β2* (*P*<0.01). Also, as *GSK3β3* and *GSK3β4* mRNA were abundantly expressed (*P*<0.01) in brain, but *GSK3β1, GSK3β2* and *GSK3β6* were relatively weak (Fig. 6).

**Figure 7:**
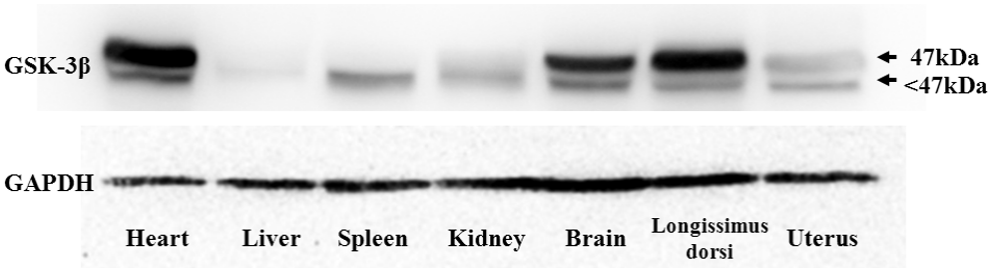
Western blotting of GSK3β proteins in goat different tissues. Equal amounts of lysate protein were immunoblotted with antibody against goat GSK3β protein. GAPDH was used as the loading control. Two bands were observed. The above band represents a larger molecular weight with major GSK3β protein expression included GSK3β1 and GSK3β5 which made up of 420-aa and 433-aa. Minor expression with lower molecular weight was observed in below, may include GSK3β3 and GSK3β6, which made up of 349-aa and 309-aa.

Western blotting was performed to determine the levels of GSK3β proteins expressed in seven tissues. GSK3β was detected in all tissues, and two bands were observed that correspond to the predicted size based on two functional domains. The major, higher molecular weight band represents a larger GSK3β protein, such as GSK3β1 or GSK3β5 (420 aa and 433 aa, respectively). The minor, lower molecular weight band, may be GSK3β3 or GSK3β6, which are composed of 349 aa and 309 aa, respectively. The results indicated that the GSK3β protein was lowly expressed in the liver. Abundant GSK3β proteins were observed in the higher molecular weight band obtained from the heart, brain and *longissimus dorsi* muscle tissues (Fig. 7). Conversely, in the spleen and kidney, relatively higher protein levels can be observed in the lower molecular weight band, indicating that splice variants have different expression levels among goat tissues.

## Discussion

In this study we cloned the *GSK3β* gene in goat and found six new splice variants. The conserved regions of the S_TKc domain, which include a glycine-rich stretch of residues located at the extreme N-terminus, a nearby lysine residue in the ATP binding region, and a conserved aspartic acid residue located at the center of the catalytic domain that is important for the catalytic activity, were identified in GSK3β1, GSK3β3, GSK3β5 and GSK3β6 [29]. Previous studies have demonstrated that GSK3β is inactivated by phosphorylation of Ser9, which leads to the dephosphorylation of glycogen synthase, a key regulatory enzyme in the process of muscle glycogen metabolism [30], [31]. GSK3β activity is dependent on the phosphorylation of Tyr216 [16]. In the present study we identified two GSK3β isoforms that lack 283 amino acids in the kinase domain: GSK3β2 and GSK3β4. These missing amino acids include two important phosphorylation sites (Ser9 and Tyr 216), key positively charged residues in the binding pocket (Arg96, Arg180, and Lys205), a binding site in GSK3β2 that would block many substrate side chains (Ser 219, Arg220, and Tyr 221), and side chains of catalytic residues Asp181 and Arg 220 that could interact with phosphorylated Tyr 216 [12], [32], [33]. The varieties of GSK3β isoforms may be associated with functional divergence. In rats, GSK3β2 is highly expressed in the nervous system, while in COS-7 cells, its phosphorylation activity on MAP1B and tau is lower than that of GSK3β1 [34]. Studies have demonstrated that the transcript lost the key phosphorylation site in the binding pocket and has little effect on the mRNA expression level of GYS1 [27]. However, the other transcripts containing Lys 205, Tyr 216 and Tyr 220 significantly reduced the mRNA expression level of GYS1 and GYS2 [27]. Moreover, two low complexity regions were found in the C-terminus of GSK3β1, GSK3β2, GSK3β4 and GSK3β5 by the SEG program. These regions may have a connection with physiological regulation, such as the finding that P38 mitogen-activated protein kinase (MAPK) inactivates GSK3β [35]. Ser 389, an inhibitory residue that directly blocks the activity of GSK3β, was not present in GSK3β3 and GSK3β6, indicating that p38 MAPK may not be able to inhibit GSK3β3 or GSK3β6 activity through phosphorylation of Ser 389 [35].

Previous studies of the adult porcine *GSK3β* show that its mRNA is abundantly expressed in the liver and testis [27], although in the goat, it is expressed at the highest levels in the heart (*P*<0.01). Our study show that *GSK3β1* and *GSK3β6* were highly expressed in *longissimus dorsi* muscle, and *GSK3β2* was highly expressed in spleen. *GSK3β4* and *GSK3β6* were highly expressed in the uterus. qRT-PCR analysis of mRNA levels in different tissues suggesting their potential functions in skeletal muscle development [36], immune system [37] and reproductive system. The high level of *GSK3β3*, *GSK3β4* and *GSK3β5* expression in goat brain tissue were similar to what was observed in previous studies, in which two *GSK3β* alternative transcripts were abundantly expressed in the mouse brain [21].

A BLAST search revealed the presence of exon 8b in goat *GSK3β*, which corresponds to exon 8b in mouse *GSK3β* [20] and porcine *GSK3β* splice variants [27]. Exon 8b in *GSK3β* showed a characteristic alignment that followed the GT-AG rule, implying that the splice sites in *GSK3β* sequences are conserved in mammals. A high expression level was detected in mouse and goat brain tissues, suggesting that the *GSK3β* that contained the exon 8b sequence has special functions in neurological tissue. However, the sequence with the inserted exon 8b in pig was expressed highest in the liver and testis. Although exon 10b in *GSK3β* has been isolated from pig [27], the deletion of the eighth exon, ninth exon and tenth exons in addition to the presence of exon 10b in *GSK3β* has never been reported. Moreover, the absence of the third, fourth, fifth and sixth exons, which is a novel feature in the genomic structure, was detected in *GSK3β2* and *GSK3β4.*

In a western blot analysis, two bands were observed corresponding to at least two protein isoforms. Four isoforms with slight differences in molecular weight-GSK3β1 (420 aa), GSK3β3 (349 aa), GSK3β5 (433 aa) and GSK3β6 (309 aa)-were present in the bands. The two smaller isoforms, GSK3β2 and GSK3β4, have no significant homology to an antibody-binding domain or an identifiable fragment in a BLAST search of established functional domain structures. They were not observed in the western blots, suggesting that the absent regions may confer different biological functions to the variant GSK3β transcripts.

## Materials and Methods

### Ethics Statement

All research involving animals was conducted according to the Regulations for the Administration of Affairs Concerning Experimental Animals (Ministry of Science and Technology, China, revised in June 2004) and approved by the Institutional Animal Care and Use Committee at the College of Animal Science and Technology, Sichuan Agricultural University, Sichuan, China under permit No. DKY-B20110807. The animals were humanely sacrificed as necessary to ameliorate suffering. Electrocutions were used as the humane form of euthanasia.

### Animals and sample collection

The Nanjiang Brown goats used in this experiment were raised under standard conditions at the Station of the Nanjiang Brown Goat Breeding Center (Nanjiang, Sichuan, China). All tissues were collected from three female goats at 120 days after birth. All tissues were collected within 30 min after slaughter and immediately frozen in liquid nitrogen.

### RNA isolation and cDNA synthesis

The total RNA was isolated from seven tissue samples (heart, liver, spleen, kidney, brain, *longissimus dorsi* muscle and uterus), which were stored in liquid nitrogen for RNA extraction. The RNA was extracted using Trizol reagent (Invitrogen, California, USA) according to the manufacturer’s instructions. The purity and quantity of the RNA were determined by the 260/280 ratio and absorbance at 260 nm, respectively. First-strand cDNA was synthesized using the Prime Script RT reagent Kit (Takara, Tokyo, Japan) as described in the manufacturer’s protocol. The corresponding cDNA was stored at −20°C.

### Cloning the goat GSK3β gene

After comparing the coding sequences of GSK3β cDNAs of *Ovis aries* (Acc. No.: NM_001129740), *Sus scrofa* (Acc. No.: JN387127), *Mus musculus* (Acc. No.: NM_019827) and *Homo sapiens* (Acc. No.: NM_002093) in Genbank, primer pairs (Table 1) were designed based on the conserved regions. The thermo cycling conditions were as follows: an initial denaturation at 95°C for 4 min followed by 35 cycles of 95°C denaturation for 30 s, 61.7°C annealing for 60 s, and 72°C extension for 90 s. A final extension was performed at 72°C for 7 min, and the reactions were stored at 4°C. The PCR products were separated by 2.5% agarose gel electrophoresis, purified using an Agarose Gel Extraction Kit (Sangon, Shanghai, China), ligated and inserted into the pMD 19-T vector (Takara, Tokyo, Japan), and transformed into *Escherichia coli* DH5α cells (Biomed, Beijing, China). Positive clones were sequenced by Invitrogen Life Technology Co., Ltd (Invitrogen, Shanghai, China).

### Molecular detection and cloning of GSK3β alternative transcripts

To obtain the alternative transcripts of goat *GSK3β,* we screened more than 100 positive clones in the process of cloning, which allowed us to visually select different *GSK3β* alternative transcripts and detect the difference among the fragments. Six new primer pairs were used to identify the specific amplicons of each *GSK3β* cDNA isoform (Table 1). The isoform-specific primer pairs were designed as follows: the forward primer of *GSK3β1* was selected based on the junction between the sixth and seventh exon, reverse primer was selected based on the junction between the eighth and ninth exon. The forward primer of *GSK3β2* was located at the second exon, and the reverse primer was across the second and seventh exon. The forward primer of *GSK3β3* was selected based on the junction between the eighth and eleventh exon, and the reverse primer was located at the 3′-UTR. The forward primer of *GSK3*β-CDS and a novel reverse primer across the second and seventh exon (different from *GSK3β2* reverse primer) were used to detect *GSK3β4*. *GSK3β5* forward and reverse pairs were selected at the site of the eighth exon and at exon 8b, respectively. *GSK3β6* forward and reverse pairs were located at the seventh exon and exon 8b, respectively (Fig. 6). Semi-quantitative reverse transcription-PCR was utilized to confirm that the primers had specifically and uniquely selected the target fragments. All the PCR bands were cut from the agarose gel for purification, sub-cloned and sequenced.

### Quantitative real-time PCR (qRT-PCR)

qRT-PCR was performed to detect the mRNA expression levels of the *GSK3β* alternative splice variants using a Bio-Rad CFX96 (Bio-Rad, California, USA). The qRT-PCR was carried out using a SYBR Green-based kit in 10 µL volumes containing 5 µL of SYBR Green Real Time PCR Master Mix (Takara, Tokyo, Japan), 0.8 µL of normalized template cDNA and 0.4 µL of each of the forward and reverse primers that were verified in the RT-PCR. The qPCR procedure was as follows: initial denaturation at 95°C for 3 min, 40 cycles of 95°C for 30 sec, alternative annealing for 30 sec, 72°C for 10 sec, and a final extension for 5 min with a temperature increment of 0.5°C/sec from 65°C to 95°C. Melting curve analysis was used to confirm specific PCR products. A cycle threshold was applied for the quantification of mRNA relative to the efficiency of *Beta-actin* expression by the comparative Ct (2^−ΔΔCt^) value method. All data are expressed as the mean ± SEM. Statistical analysis was performed using one-way ANOVA with the SAS Statistical Analysis System (SAS Institute Inc., NC, USA).

### Bioinformatic sequence analysis

The molecular weight and isoelectric point (pI) were calculated by EditSeq 7.10 (DNAstar, Inc. Wisconsin, USA). ClustalW (http://www.ebi.ac.uk/clustalw/) was used for the multiple sequence alignment. The open reading frame was translated and BLAST searched using the NCBI ORF Finder (http://www.ncbi.nlm.nih.gov/gorf/gorf.html). The domain structure of the GSK3β proteins was searched by BLAST and analyzed with the SMART (http://smart.embl.de/) server.

### Western blotting

Total proteins were extracted from different tissues using a Tissue or Cell Total Protein Extraction Kit (Sangon, Shanghai, China) and normalized with a BCA Protein Assay Kit (Sangon, Shanghai, China). The sample and buffer (Beyotime, Shanghai, China) were mixed well, and 20 µg of total protein was loaded per lane in a precast 10% polyacrylamide gel. After SDS-PAGE, the proteins were transferred from the gel to a PVDF membrane. The membranes were blocked with blocking buffer (Beyotime, Shanghai, China) and incubated with primary antibodies (GSK3β Rabbit mAb 27C10, Cell Signaling Technology, Inc, MA, USA) overnight at 4°C. After the membranes were washed with TBST, they were incubated with the secondary antibody (HRP-labeled goat anti-rabbit IgG H+L A0208, Beyotime, Shanghai, China) for 2 h at 37°C. After washing the membrane with TBST and TBS, the proteins were visualized using an ECL detection system (BeyoECL Plus, Beyotime, Shanghai, China). The GAPDH (AG019, Beyotime, Shanghai, China) protein was utilized as an internal control.

## Supporting Information

Figure S1 **Design of isoform-specific primer pairs for qRT-PCR.** Black arrows label the isoform-specific primer pairs that used in qRT-PCR. Red frame label the primer pairs that used in RT-PCR to facilitate visualization of the RT-PCR results.
(TIF)
