# Supplementary figures and images for: Multiple Alternative Splicing and Differential Expression Pattern of the Glycogen Synthase Kinase-3β (GSK3β) Gene in Goat (Capra hircus)
Source: PLoS One. 2014 Oct 15;9(10):e109555. doi: 10.1371/journal.pone.0109555 (PMC4198110; doi:10.1371/journal.pone.0109555)

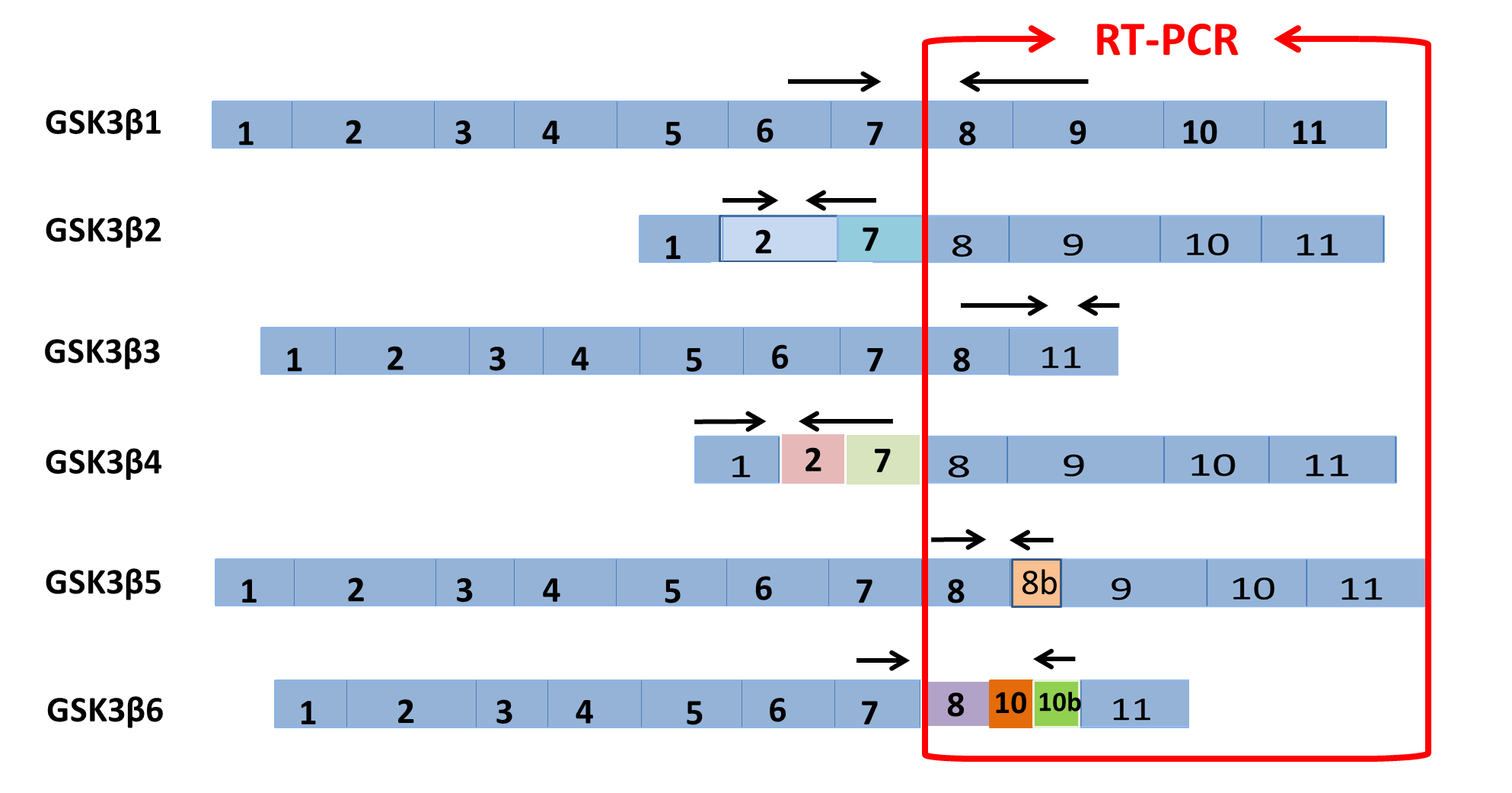

Supplement: Figure S1 — Design of isoform-specific primer pairs for qRT-PCR. Black arrows label the isoform-specific primer pairs that used in qRT-PCR. Red frame label the primer pairs that used in RT-PCR to facilitate visualization of the RT-PCR results. (TIF) [file pone.0109555.s001.tif]
